# The Role of Recombinant Proteins and Growth Factors in the Management of Diabetic Foot Ulcers: A Systematic Review of Randomized Controlled Trials

**DOI:** 10.1155/2020/6320514

**Published:** 2020-07-11

**Authors:** Elahe Mahdipour, Amirhossein Sahebkar

**Affiliations:** ^1^Department of Medical Biotechnology and Nanotechnology, Faculty of Medicine, Mashhad University of Medical Sciences, Mashhad, Iran; ^2^Halal Research Center of IRI, FDA, Tehran, Iran; ^3^Biotechnology Research Center, Pharmaceutical Technology Institute, Mashhad University of Medical Sciences, Mashhad, Iran; ^4^Neurogenic Inflammation Research Center, Mashhad University of Medical Sciences, Mashhad, Iran; ^5^Polish Mother's Memorial Hospital Research Institute (PMMHRI), Lodz, Poland

## Abstract

**Background:**

Recombinant proteins and growth factors are emerging therapies for diabetic foot ulcers. Despite several clinical reports, there has been no comprehensive and systematic assessment of the totality of clinical evidence on the efficacy and safety of recombinant proteins and growth factors in diabetic foot ulcers. We tried to address this gap through an updated systematic review of randomized controlled trials (RCTs).

**Methods:**

PubMed, the Cochrane Library, Scopus, Embase, and Google Scholar databases were searched, and RCTs on the efficacy of recombinant proteins and growth factors in the treatment of cutaneous wounds in diabetic patients were selected. The literature search and assessment were performed by two independent reviewers. Methodological quality of studies was appraised using the Jadad scale.

**Results:**

We identified 26 RCTs involving diabetic patients with ulcer that evaluated the effectiveness of platelet-derived growth factor, epidermal growth factor, fibroblast growth factor, granulocyte colony-stimulating factor, vascular endothelial growth factor, erythropoietin, transforming growth factor, talactoferrin, and rusalatide acetate. The main primary outcome was complete healing though different indices were employed to define this such as wound closure, granulation tissue formation, or complete reepithelialization. Few studies had a follow-up period to report any recurrence and amputation rate. No adverse effect was reported due to the intervention.

**Conclusion:**

Overall, there is a greater agreement on the effectiveness of EGF to enhance the healing of diabetic ulcers. Nevertheless, extant evidence is lacking for other agents since few trials have been conducted for most of the growth factors and available studies are heterogeneous in their methodologies.

## 1. Introduction

Wound repair is a complex and dynamic process composed of four overlapping healing phases of hemostasis, inflammation, tissue formation, and tissue remodeling [1]. Various residence or migratory cell types are involved in the production of regulatory mediators and extracellular matrix to make new tissue. Cytokines and growth factors play a major role in this networking, and any growth factor dysfunction results in impaired wound repair such as diabetic wound [1]. Impaired healing of the cutaneous wound is one of the most important complications of diabetes. A meta-analysis in 2017 reported the global prevalence of diabetic ulcer to be 6.3% (95% CI: 5.4-7.3%), which was higher in males than in females [2]. The management of diabetic foot ulcer imposes a significant financial burden to patients and societies. Furthermore, the current standard of care for diabetic foot ulcer that comprises infection control, debridement, local wound care, and offloading is not enough for most diabetic wounds to heal [3]. This increases the incidence of amputation. Under physiological condition, after an injury, platelet-derived growth factor (PDGF) is released by activated platelets and plays a role in different stages of healing. PDGF is one of the first factors that received approval from the FDA, and its efficacy has been evaluated in randomized controlled trials (RCTs) [4]. This factor acts as a mitogen and chemoattractant of regulatory cells to the wound environment [5]. Moreover, PDGF stimulates the production of other growth factors such as vascular endothelial growth factor (VEGF) and transforming growth factor *β* (TGF-*β*) [5]. VEGF is a famous regulator of neoangiogenesis that should occur in newly formed tissue at a wound site [5]. TGF-*β* family has several members that are involved in several steps such as inflammation, angiogenesis, reepithelialization, and collagen production [5]. Epidermal growth factor (EGF) is another main wound modulator that is involved in cell migration and proliferation. In conjunction with fibroblast growth factor (FGF), EGF enhances reepithelialization [5]. FGF has a big family of 23 members from which FGF2, FGF7, and FGF10 have important roles in cutaneous wound repair. FGF2 increases the migration of keratinocytes during reepithelialization and stimulates fibroblast migration and collagen production [5]. Chronic wound fails to produce functional FGF2 [6], which is a reason for the utilization of FGF2 in clinical studies to enhance closure of chronic wounds such as diabetic ulcers.

Owing to their significant role during wound repair, exogenous application of growth factor has emerged as a promising therapeutic approach and has been investigated in several clinical studies. Most of these studies suffer a number of methodological flaws. They are inconsistent in several features such as the applied standard of care, type of wound dressing, and the application of antibiotics. While the wound closure is the main outcome, studies have different definitions of that. Some considered the reepithelialization as the complete healing while others not. These important issues are generally disregarded in systematic reviews. Herein, we report an updated systematic review of randomized controlled trials investigating recombinant proteins or growth factors for the purpose of wound healing. Besides discussing the findings, we have carefully appraised the evaluation methods and possible variables that affect the outcomes of studies.

## 2. Methods

### 2.1. Search Strategy

A systematic literature search was performed in PubMed, the Cochrane Library, Scopus, Embase, and Google Scholar databases. The search was formulated using the following terms: recombinant, protein OR peptide OR growth factor OR cytokine AND therapy, diabetic OR diabetes, skin OR cutaneous, wound OR injury OR feet OR foot OR ulcer∗ AND clinical OR trial∗ OR random∗. The references of relevant studies were manually searched to avoid missing any relevant article. The search was performed from inception to April 16, 2019. Only studies published in English language were considered. The review was conducted according to the Preferred Reporting Items for Systematic Reviews and Meta-Analysis (PRISMA) guideline. Inclusion criteria consisted of RCTs, both placebo- and active-controlled trials, patients with type 1 or 2 diabetes and cutaneous wound, and topical administration of growth factors or recombinant proteins. Exclusion criteria were nonoriginal studies, nonrandomized or uncontrolled trials, studies on nondiabetic wounds, studies using PRP (platelet-rich plasma) or comparing growth factors with skin substitutes, and studies that applied growth factor-expressing vectors to enhance the growth factor level.

### 2.2. Study Procedure

The literature searches and assessments were performed by two independent reviewers and in the instance of uncertainty, a third reviewer was consulted. Various amounts of information were extracted from studies such as author name, year of publication, study design, blinding method, number of involved patients, subjects' demographic information, Hb1Ac level, type and grade of wounds, wound size, antibiotic administration in the course of intervention, type of intervention and the related dose of the active agent, treatment duration, administration method (injection or topical cream/gel), type of control group (active or placebo), type of dressing and offloading, primary outcome, duration to achieve complete healing, criteria to define complete healing, recurrence rate, amputation rate, follow-up period, and adverse effects. We also looked for any data related to the patient's quality of life; however, no information was found. The study protocol has been registered in the international prospective register of systematic reviews (PROSPERO). The registration number is CRD42020143221.

### 2.3. Analysis of the Selected Articles

The included articles were critically appraised using the Jadad scale for reporting RCTs [7]. Furthermore, a purpose-built analytical scale was created for the review, looking for details on the strategies used to conduct trials, and the criteria to evaluate the effectiveness and safety of treatments.

## 3. Results

**Figure 1:**
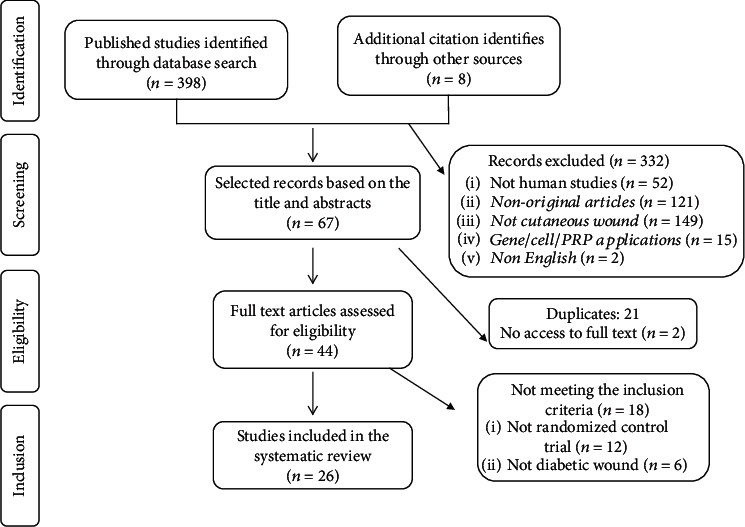
Study flow diagram for identification of eligible studies to review.

From a total of 406 identified articles, only 26 studies were eligible to be included in the final systematic review (Figure 1). In the first step of analysis, a total of 332 studies were excluded after assessment of titles and the abstracts for the following reasons: studies were performed on nonhuman models (*n* = 52), review articles (*n* = 121), not being published in English (*n* = 2), and a large number of studies (*n* = 149) were related to conditions other than cutaneous injuries. Since the composition of PRP is undefined and inconsistent, we also excluded studies that used PRP or growth factor-expressing vectors as the intervention (*n* = 15). From the remaining articles, 23 studies were excluded because of the duplication or no access to the full text. Full-text evaluation resulted in the further exclusion of 18 studies for not being conducted as RCT (*n* = 12) or treating injuries other than diabetic ulcers such as pressure ulcers, venous ulcers, and burns (*n* = 6). The resulting 26 eligible studies were subjected to review. Studies were divided into 5 groups that evaluated the safety and efficacy of PDGF (*n* = 8), EGF (*n* = 5), FGF (*n* = 4), G-CSF (*n* = 4), and other protein and growth factors including VEGF (*n* = 1), erythropoietin (*n* = 1), TGF-*β* (*n* = 1), talactoferrin (*n* = 1), and rusalatide acetate or Chrysalin® (*n* = 1).

### 3.1. PDGF

Eight RCTs [8–15] were mined for information to show the effectiveness of PDGF for diabetic wound repair from which two studies were conducted as phase III trials [8, 14]. Since the study design will directly affect the reliability of results, we considered various criteria that may affect the outcomes such as the dose and duration of treatment (Table 1). All studies applied PDGF in the form of a topical gel (mainly formulated with sodium carboxymethyl cellulose), however, in different concentrations of 30 or 100 *μ*g/g of gel, in the form of 0.01% PDGF gel, or in one study as 7 *μ*g PDGF/cm^2^ of ulcer. Studies compared the results with placebo control except for three studies that used active controls composed of KY Jelly [13], hyperbaric oxygen and antiseptics [12], and TheraGuaze [15]. Treatment duration was around 20 weeks for most studies except two RCTs that performed treatment for 10 weeks [12, 13]. Only 2 studies reported that they had a posttreatment follow-up of 3-6 months to evaluate the secondary outcomes of recurrence and amputation [8, 10]. The studied wounds were almost at the same grade and equal to Wagner's grade II or III, except one study that treated Wagner grade I wound [10]. Studies are in agreement with the type of dressing used for patients, and except for one study, offloading was in place. Three studies did not prescribe antibiotics during the treatment period. The HbA1c was fairly under control for most studies.

The efficacy of PDGF was mainly evaluated based on wound closure (Table 2). Considering the fact that the wound closure can be achieved with contraction and granulation, tissue formation then will be stabilized by reepithelialization. Only three studies considered reepithelialization as complete healing [10, 14, 15]. One study mentioned complete wound contraction as main outcome [12]. Studies were also evaluated for any reports of possible confounding factors such as sex, HbA1c, wound size, and offloading. For most studies, no data were mentioned regarding these confounders. However, three studies found a positive correlation between offloading and complete healing [8, 10, 11]. Two studies found a negative correlation between wound size and healing [10, 14], while no wound size correlation was reported in three studies [8, 11, 12]. The effect of HbA1c was only assessed by two studies which found no correlation [8, 10]. No information was available from studies regarding the amputation rate. Recurrence rate was only reported by two studies, in which there was no significant difference between PDGF- or placebo-treated group [8, 9]. Four studies did not find the healing effect of PDGF significant from which one study concluded that the PDGF is not recommended for Wagner grade I wound [10]. The other three studies did not find the significant healing improvements compared with groups that received standard wound care [11], KY Jelly [13], or TheraGuaze [15]. However, the remaining four trials found higher and faster wound repair after PDGF application [8, 9, 12, 14].

### 3.2. EGF

Five randomized controlled trials (one in phase III) assessed the efficacy of recombinant EGF in improving the healing of diabetic ulcers [16–20] (Tables 3 and 4). EGF was used as intralesional injections [16, 17] or as a topical cream/gel [18–20]. Placebo control was used; however, in one study, the Betadine dressing was used for the control group [18]. Another study used Actovegin (calf blood extract) for the control group and Actovegin plus EGF for the treated group [19]. Wounds from various Wagner grades were treated for a duration of 8-15 weeks. All studies found significant improvement of wound healing in the EGF group. Granulation tissue formation and reepithelialization were mentioned as the mechanism of healing. No data was available related to the effect of cofounders; just one study reported no influence of sex on the outcome. Only 2 studies had a follow-up period and reported the number of cases with amputation [17, 19]. However, the effect of EGF in decreasing or increasing the chance of amputation was not clear. One of these studies also reported 2 cases of recurrence in the placebo group [17].

### 3.3. FGF

We found four RCTs that evaluated the healing potential of FGF on diabetic ulcers [21–24] (Tables 5 and 6). Liquid FGF was used, for example, in the form of a spray in different concentrations of 40 U/cm^2^, 100 U/cm^2^, 500 ng/wound, 1 *μ*g/wound, 50 *μ*g/wound, and 500 *μ*g/wound on a daily basis. One of these studies applied the acidic form of FGF (aFGF) or FGF1 and did not find significant improvement in the healing process during a period of 60-day treatment [22]. However, they had 4 arms in their study, one received only aFGF, the second group received EGF, and the third group received a combination of FGF and EGF which were compared with the placebo-treated group. They reported that the healing achieved in a shorter period in patients received the combination therapy and EGF alone compared with placebo [22]. Tan et al. compared the efficacy of bFGF with aFGF after 6 weeks of application and did not found any significant difference in the healing potential of acidic or basic forms of FGF [21]. The other two studies applied bFGF topically, and one of them showed that compared with placebo, after 8 weeks of therapy, bFGF can significantly decrease the wound size only at high-dose (500 *μ*g) application [24]. Only two RCTs defined the healing outcome as the formation of granulation tissue and new epithelial formation [22, 24]. No information was available from studies regarding the confounder consideration or evaluation of amputation and recurrence rate. Treated wounds were from different Wagner grades I-III. No data regarding the posttherapy follow-up was found.

### 3.4. G-CSF

The main objective of trials that conducted G-CSF therapy was to enhance the immune reaction to eradicate wound infection (Table 7). Therefore, the main outcomes to evaluate were the microbial burden, cellulitis resolution, duration of hospitalization, and antibiotic administration. Three trials [25–27] used 5 *μ*g/kg G-CSF as a systemic injection for 7-10 days, and just one study found a significant effect of G-CSF on the quicker resolution of cellulitis, shorter hospitalization, and shorter duration of antibiotic application [25]. Kastenbauer et al. [26] found more reduction in ulcer volume in the G-CSF-treated group while the effect on cellulitis and amputation rate was not substantial. The fourth trial [28] which used 263 mg of G-CSF daily for 21 days reported no significant difference in healing rate and infection status of Wagner grade III/IV diabetic wounds [28]. However, they found a fewer amputation rate in the G-CSF treated subjects (*p* = 0.03) [28] (Table 8).

### 3.5. Other Growth Factors and Recombinant Proteins

A phase I randomized controlled trial evaluated the safety and efficacy of recombinant VEGF to treat grade 1A diabetic wounds in 55 patients for a duration of 6 weeks and a follow-up period of 7-12 weeks [29] (Table 9). They reported a positive but nonsignificant healing trend in VEGF-treated patients [29]. No mechanism of healing was mentioned, and no confounders were stated to be evaluated in the study. However, a fewer recurrence rate was found in the VEGF-treated group (not significant) [29] (Table 10).

The effectiveness of erythropoietin on diabetic wound closure was studied by a phase IIa RCT, in which Wagner grade I/II wounds were treated with 30 IU/kg/week of erythropoietin subcutaneously for a duration of 12 weeks [30] (Table 9). The result of the study represented not a significant increase in the percentage of patients achieving complete healing compared with the placebo control arm. No further information regarding the mechanism of healing and the confounding effect of variables was available from the study [30] (Table 10).

Talactoferrin, which is the recombinant human lactoferrin, was used to treat diabetic ulcers in phase I/II RCT. For a 12-week period, a topical 2.5% or 8.5% talactoferrin gel was applied twice daily [31] (Table 9). The active arm showed a trend toward enhanced healing over placebo (*p* = 0.09) [31] (Table 10). Another RCT study assessed the potential of Chrysalin® to enhance the healing of diabetic ulcers [32] (Table 9). Chrysalin® which is a Thrombin peptide was applied at 1 or 10 *μ*g concentrations to treat diabetic ulcers at different Wagner grades (I-III) for 20 weeks. This treatment resulted in an increased mean closure rate and decreased time to complete wound reepithelialization in a dose-dependent manner [32] (Table 10).

TGF-*β*2 that is one of the main cytokines involved in wound repair was used at 0.05 *μ*g/cm^2^ and 0.5 *μ*g/cm^2^ in an RCT [33]. The study design was composed of 4 experimental groups receiving topical placebo collagen sponge, topical collagen sponge loaded with 0.05 *μ*g/cm^2^, or 0.5 *μ*g/cm^2^ of TGF-*β*2 and finally a group that received standard care including sharp debridement and weight offloading (Table 9). The results claimed that compared with placebo, a higher percentage of patients that received TGF-*β*2 at doses of 0.05 *μ*g/cm^2^ (*p* = 0.046) and 0.5 *μ*g/cm^2^ (*p* = 0.025) or standardized care treatment (*p* = 0.009) achieved healing. TGF-*β*2 at high dose reduce the median time to complete wound closure (*p* = 0.03) (Table 10). This study reported a negative correlation between wound size and the rate of complete healing, but no correlation for sex and HbA1c level [33] (Table 10).

### 3.6. Adverse Events

Various adverse effects were reported by studies from which we have summarized the more frequently reported effects in Table 11 including pain, erythema, edema, infection, and cellulitis. However, none of the adverse effects were proved to be drug-related except for EGF events such as dizziness, shivering, and chills observed more frequently in the EGF-treated groups, which re apparently dose-dependent (Table 11).

### 3.7. Quality Assessment

The quality of RCTs was assessed using Jadad score and is summarized in Table 12 The mean of Jadad score was 3.15 ± 1.04. Most studies have a shortage in reporting the randomization or blinding methods. Only six studies reported evidently their randomization protocol [9, 13, 16, 20, 25, 33], and four of them [16, 20, 25, 33] were also defined by their blinding methods. Six RCTs did not mention that they have conducted a blinded trial [11–13, 15, 18, 27]. And finally, all studies were clear for the withdrawal and patient's dropouts.

## 4. Discussion

In this systematic review, we have summarized the results from RCTs that were aimed at treating diabetic ulcers by using recombinant proteins and growth factors. Diabetic foot ulcers are one of the most serious health complications with a worldwide prevalence of 6.3% [34]. In fact, 15-25% of diabetic patients will suffer a diabetic foot ulcer during their lifetime [35], which can lead to serious outcomes such as amputation. Consequently, a safe and effective therapy is highly demanded. Growth factors and cytokines are major regulatory factors during wound repair, and their dysfunction results in impaired healing and formation of chronic wounds [5, 36]. Therefore, several experimental and clinical studies have attempted to regulate wound repair through exogenous applications of these factors. Despite their significant role in healing, clinical studies have provided unequivocal results as to the superior efficacy of recombinant growth factors in treating diabetic ulcers. In an attempt to obtain a conclusive result, we systematically reviewed RCTs as the highest level of evidence with respect to the methodological aspects and the obtained results.

PDGF with commercial names of Becaplermin® and Regranex® is approved by the FDA for the treatment of diabetic ulcers [9]. As Table 2 shows, not all trials reported in this review are in agreement with positive effectiveness of PDGF to improve healing of the diabetic wound. The meta-analysis of these trials suggested the benefits of PDGF in diabetic ulcers [37, 38]. However, these reviews did not include studies that compared PDGF with active controls, which can be a limitation of their analysis. Here, we observed the studies that compared PDGF with active controls such as TheraGuaze [15] and did not found the superiority of PDGF treatment. For safety issue, studies did not report an adverse event related to the drug. Moreover, in 1998, Smiell [39] and the Becaplermin study group reported an overview of the safety of Becaplermin® gel based on six RTCs. Besides adverse events such as infection, cellulitis, osteomyelitis, and erythematous rash, they also evaluated the incidence of cardiovascular, respiratory, musculoskeletal, and central nervous system disorders. The conclusion suggests that Becaplermin® gel is possibly safe for the treatment of diabetic ulcers [39]. A cohort comprising 1622 Becaplermin initiator and 2809 matched comparators studied the risk of cancer following treatment with Becaplermin® or PDGF. The results showed no increased risk of cancer with PDGF (hazard ratio, 1.2; 95% CI, 0.7-1.9) [40].

Among other growth factors, FGF application has been associated with controversial results. It seems that FGF is effective when administered at high doses (500 *μ*g or 100 U/cm^2^). Tan et al. performed a pharmacokinetic study before conducting trial. They showed that after topical application of FGF in rabbits, the plasma level of FGF rapidly increased and reached a peak at half an hour and then decreased to normal level after 3 hours [21]. Although the result from this animal model showed FGF to be nontoxic, such pharmacokinetic studies are valuable if considered in clinical trials. Unlike FGF, almost all trials that evaluated the efficacy of EGF reported a significant improvement. Previous meta-analysis and reviews also proposed the beneficial effect of EGF for the healing of diabetic wounds [38, 41]. Cost-effectiveness analysis of EGF application in patients with Wagner grade III/IV diabetic wound found EGF as a more effective therapeutic option than conventional therapy. They reported 39% less amputation in EGF-treated patients [42]. RCTs that conducted G-CSF therapy apparently demonstrated no benefits in terms of infection eradication (Table 8). However, G-CSF may have more benefits as it is shown to accelerate angiogenesis and wound healing [43]. Unfortunately, the follow-up period of available studies is not long enough to evaluate any improvement in wound repair. The effectiveness of other growth factors and recombinant proteins mentioned in this review is hard to be concluded as results are from single studies and for some, such as erythropoietin, a small number of patients were studied [29]. Although demonstrated just by one RCT, Chrysalin® and TGF-*β*2 could significantly improve healing of diabetic ulcers [32, 33]. Chrysalin®, also known as rusalatide acetate or TP 508, is a peptide that can bind to cell surface receptors and activates several signaling such as nitric oxide [44]. As the diabetic wound is deficient in nitric oxide [45], Chrysalin® can be beneficial for wounds. However, Chrysalin® is in the list of 2014 discontinued dermatological drug apparently for financial reasons [46].

## 5. Conclusion

Despite the promising effectiveness of some growth factors such as EGF, the number of controlled trials is small and most of them do not have good methodological quality. Almost half of the trials for PDGF and FGF were not blinded which might be a source of performance and detection bias (Table 12). Blinding has been considered very important to produce more consistent results [47]. This might be accounted as a reason for disagreement in the results of trials. Moreover, various possible confounders are present in studies such as wound size, HbA1c, type of dressing, sex, age, and offloading. Studies considered their groups to be approximately the same for age and sex. However, differences in wound size has been wide and few trials have analyzed the effect of this covariate on the healing rate. Offloading has been shown to have a positive effect on healing, while in most studies, offloading has not been offered to all of the patients. This can be a critical source of variation in results. Ma et al. [10] in their study used offloading for all patients and have shown that offloading alone can result in excellent healing results. Dressing type is another important and effective covariate that should be taken into account when the results from different studies are compared with each other. Finally, as wound healing is a complicated process that comprises of various growth factors and cytokine which acts in a network, it is not surprising that single growth factor therapy ends in not remarkable benefits. For example, Becaplermin® has got its approval a long time ago; however, this drug has not been used widely in everyday practice. In this regard, the conduction of RCTs assessing the different combinations of growth factors is highly demanded.

## Figures and Tables

**Table 1:** Characteristics of RCTs evaluated PDGF safety and effectiveness.

Ref	Study	Intervention	Type of control	Size and the old of the wound	# of patients	Antibiotics application during the treatment period (if needed)	Baseline HbA1C	Types of wound and grade of wound	Dressing type	Offloading	Treatment duration	Follow up period posttherapy
[8]	Phase III RCT	Becaplermin® gel (Regranex) 100 and 30 *μ*g/g vehicle gel once daily	Placebo	>2cm^2^ for a period of at least 8 weeks	382	Y	6.5-7.2	Stage III or IV (IAET guide)	Moist saline-soaked gauze dressings	Y	20 weeks	3 months
[9]	RCT	30 *μ*g PDGF per g of gel once a day	Placebo	1-100 cm2 at least 8-week duration	118	N	NM	NM	Non adherent saline soaked gauze	Y	20 weeks	NM
[10]	RCT	PDGF gel once daily	Placebo hydrogel	1-16 cm^2^	46	N	Y	Wagner grade I	Saline moistened gauze and nonadherent wound dressing	Y	4 months	6 months
[11]	RCT	0.01% rhPDGF-BB gel once a day	Standard wound care	14.6 ± 13.2 at least 4-week duration	20	N	8.05 ± 0.84	Wagner's grade II	Moist saline and casting	Y	20 weeks	NM
[12]	RCT	PDGF gel 7 *μ*g/cm^2^ of ulcer per day	Two active controls: antiseptics and hyperbaric oxygen therapy	8-week duration	60	Y	NM	Equals to Wagner grade II, III	Saline moistened gauze	NM	10 weeks	NM
[13]	RCT	rhPDGF gel 0.01%	Active: KY Jelly (Ethnor)	26-30 cm^2^ at least 4-week duration	50	Y	NM	IAET stage III and IV	Moist dressing	Y	10 weeks	NM
[14]	RCT (phase III)	0.01% PDGF gel containing 100 *μ*g/g	Placebo	1-40 cm2 at least 4 weeks	111	Y	<12%		Moist saline gauze	Y	20 weeks	NM
[15]	RCT	Regranex (PDGF) 0.01% plus TheraGuaze	Active (TheraGuaze)	1-8 cm^2^	32	NM	<10%	Wagner grade I/II	TheraGuaze	Y	NM	Study period: 20 weeks

PDGF: platelet-derived growth factor; Y: yes; N: no; NM: not mentioned; IAET: International Association of Enterostomal Therapy.

**Table 2:** Outcomes of RCTs that evaluated PDGF safety and effectiveness.

Ref	Type of growth factor	Wound closure	Mean time to heal in treatment groups	Mechanism mentioned as complete healing		Confounders				Further outcomes	
				Granulation tissue	Reepithelialization	Sex	Baseline HbA1c	Wound size	Offloading	Recurrence rate	Amputation rate
[8]	PDGF-Becaplermin®	50%, 35%, and 36% of complete healing in 100 *μ*g/g Becaplermin® gel and placebo and 30 *μ*g/g Becaplermin® gel, respectively	86 days for 100 *μ*g/g Becaplermin® gel (decreased time by 32%)	NM	NM	NM	N	N	Y(+)	30% in all groups	NM
[9]	rhPDGF-BB gel	48% complete healing in the PDGF group compared with 25% in the placebo group *p* = 0.01	30 days in the PDGF and 40 days in the placebo group. *p* = 0.01	NM	NM	NM	NM	NM	NM	26% in PDGF treated versus 46%	NM
[10]	Topical PDGF	52% of healing in the test group versus 57% of healing in the control group (not significant)	16 weeks	NM	Y	NM	N	Y(-)	Y(+)	NM	3 cases in total
[11]	rhPDGF-BB gel	All ulcers in both groups had healed by the end of the study period	50.10 ± 23.38 days 41.8% reduction in healing time (*p* = 0.02)	NM	NM	NM	NM	N	Y(+)	NM	NM
[12]	PDGF	Percentage of patients with complete wound contraction was significantly (*p* = 0.03) higher in the PDGF group compared to the other groups	6.75-7.6 weeks Not significant	NM	NM	NM	NM	N	NM	NM	NM
[13]	PDGF	18 (72%) ulcers had healed in the control group and 15 (60%) in the test group (*p* > 0.05). Three ulcers in the control group showed >75% reduction in size compared to 2 in the test group (*p* > 0.05).	10 weeks	NM	NM	NM	NM	NM	NM	NM	NM
[14]	PDGF gel	A significantly higher (*p* < 0.01) percentage of patients in the rhPDGF-based gel-treated group achieved complete healing	46 days (*p* < 0.001)	NM	Y	NM	NM	Y(-)	NM	NM	NM
[15]	PDGF	The rates of wound closure with TheraGauze and TheraGauze + Becaplermin® were 0.37 and 0.41 cm2/week, respectively (*p* = 0.34)	12 weeks	NM	Y	NM	NM	NM	NM	NM	N

PDGF: platelet-derived growth factor; Y: yes; N: no; NM: not mentioned.

**Table 3:** Characteristics of RCTs that evaluated EGF safety and effectiveness.

Ref	Study	Intervention	Type of control	Size and the oldness of the wound	No. of patients	Antibiotic application during the treatment period (if needed)	Baseline HbA1C (%)	Types of wound and grade of wound	Dressing type	Offloading	Treatment duration	Follow-up period posttherapy
[16]	RCT	Thrice-per-week intralesional application of 75 *μ*g rhEGF	Placebo	>2 cm^2^	31	Y	NM	Any grade	Antimicrobial dressing with ionic silver	Y	8 weeks	NM
[17]	RCT	EGF (75 or 25 *μ*g) three times per week and standard good wound care	Placebo	*>*1 cm^2^	149	Y	Y	Wagner's grade III or IV	Saline-moistened gauze	Y	8 weeks	12 months
[18]	RCT	Topical application of beta urogastrone (rhEGF) gel. It was applied as a thick layer	Betadine dressing	2-50 cm^2^ in the area	50	NM	NM	Wagner grades I and II	Dry sterilized gauze	NM	8 weeks	NM
[19]	RCT	Group 1 (control) was treated with Actovegin 5% cream (Actovegin), group 2 with Actovegin plus 0.02% (wt/wt) hEGF, and group 3 with Actovegin plus 0.04% (wt/wt) hEGF	Placebo	NM	61	NM	<12%	Wagner grades I and II	Saline dressing	NM	12 weeks	24 weeks
[20]	RCT phase III	rhEGF 150 *μ*g/g gel	Placebo	2-50 cm^2^ More than 2-3 weeks old	60	NM	NM	Wagner grades I and II	NM	NM	15 weeks	NM

EGF: Epidermal growth factor, Y: yes, N: no, NM: not mentioned.

**Table 4:** Outcomes of RCTs that evaluated EGF safety and effectiveness.

Ref	Type of growth factor	Wound closure	Mean time to heal in treatment groups	Mechanism mentioned as complete healing		Confounders				Further outcomes	
				Granulation tissue	Reepithelialization	Sex	Baseline HbA1c	Wound size	Offloading	Recurrence rate	Amputation rate
[16]	EGF	More complete healing in the rhEGF group (*p* = 0.033); decreased in area size (*p* = 0.049); and more epithelial islands in the wound bed were present (*p* = 0.025)	8 weeks	Y	Y	NM	NM	NM	NM	NM	NM
[17]	EGF	Granulation tissue covering ≥50% of the ulcer at 2 weeks was achieved by more cases in the EGF groups (*p* = 0 · 000015). Shorter time to complete healing in the 75 *μ*g group (*p* = 0.006)	3 weeks	Y	NM	NM	NM	NM	NM	2 cases in the placebo group	29 cases in all groups
[18]	EGF	Reduced seropurulent discharge in the EGF group *p* = 0.0495 and serous discharge *p* = 0.009. More granulation tissue *p* = 0.041. More complete healing in the EGF group *p* = 0.007	17.2 ± 1.3 (*p* = 0.01)	Y	NM	NM	NM	NM	NM	NM	NM
[19]	EGF	More cases with complete healing in the 0.04% hEGF group. Patients in the 0.04% hEGF group also healed more quickly than those in the other groups (*p* = 0.0003). No significant difference in healing time between the 0.02% hEGF and control groups	6 weeks in the 0.04% hEGF group (*p* = 0.0003)	Y	Y	N	NM	NM	NM	NM	2 cases in placebo and 2 in 0.02% hEGF groups
[20]	EGF, REGEN-D150	For wounds >6 cm^2^ in size treatment resulted in more healing (*p* < 0.002). A reduced healing time in the EGF group. At the end of 10 weeks, 69% of wounds healed versus 21% in placebo control	9 weeks	Y	Y	NM	NM	NM	NM	NM	NM

EGF: epidermal growth factor; Y: yes; N: no; NM: not mentioned.

**Table 5:** Characteristics of RCTs that evaluated FGF safety and effectiveness.

Ref	Study	Intervention	Type of control	Size and the oldness of the wound	No. of patients	Antibiotic application during the treatment period (if needed)	Baseline HbA1C (%)	Types of wound and grade of wound	Dressing type	Offloading	Treatment duration	Follow-up period posttherapy
[21]	RCT	Topical rhaFGF (liquid) and rhbFGF at a dose of 100 U/0.1 mL/cm^2^	Active (bFGF)	>2 cm in diameter At least 8 weeks	139	N	NM	NM	Sterile cotton dressings without antibiotics	NM	6 weeks	NM
[22]	RCT	4 groups: hEGF (liquid) at 40 IU/cm^2^ and aFGF at 40 AU/cm^2^ or hEGF at 40 IU/cm^2^ or topical aFGF 40 AU/cm^2^ or the wound was cleaned with normal saline only	Placebo	>3 cm^2^ At least 12 weeks	199	N	NM	Grade II Wagner	NM	NM	60 days	NM
[23]	RCT	Liquid bFGF spray, 500 ng-100 ng/wound	Placebo	> 0.5 cm More than a year	17	Y	7.1-7.9	Wagner grades I–III	Sterile petrolatum impregnated gauze (no antiseptic)	Y	18 weeks	NM
[24]	RCT	0.001% bFGF (50 *μ*g) and 0.01% FGF (500 *μ*g) spraying once a day	Placebo	900 mm2 or less	150	Y	10-16	Wagner grade II	Silicone gauze	Y	8 weeks	NM

FGF: fibroblast growth factor; Y: yes; N: no; NM: not mentioned.

**Table 6:** Outcomes of RCTs that evaluated FGF safety and effectiveness.

Ref	Type of growth factor	Wound closure	Time to heal	Mechanism mentioned as complete healing		Confounders				Further outcomes	
				Granulation tissue	Reepithelialization	Sex	Baseline HbA1c	Wound size	Offloading	Recurrence rate	Amputation rate
[21]	rhaFGF and rhbFGF	Healing in rhbFGF by 6-week treatment. No significant difference between the healing potential of bFGF and aFGF	42 days to complete healing in almost 50% of cases	NM	NM	NM	NM	NM	NM	NM	NM
[22]	Liquid aFGF and EGF	Healing in shorter period in the combination group (*p* < 0.01) and in EGF-treated group (*p* < 0.05) compared with the control group	36-47 days in combination-treated group versus control	Y	Y	NM	NM	NM	NM	NM	NM
[23]	Liquid bFGF	No significant difference	Mean healing time: 9.3 weeks for the bFGF and 5.8 weeks for the control group	NM	NM	NM	NM	NM	NM	NM	NM
[24]	bFGF	The area of ulcer decreased by 57.5%, 72.3%, and 82.2% in the placebo, 0.001% in the bFGF, and 0.01% in the bFGF groups, respectively, and differences were significant between the 0.01% bFGF and placebo groups (*p* = 0.025)	NM	Y	Y	NM	NM	NM	NM	Approximately 10% in all groups	NM

FGF: fibroblast growth factor; Y: yes; N: no; NM: not mentioned.

**Table 7:** Characteristics of RCTs that evaluated G-CSF safety and effectiveness.

Ref	Study	Intervention	Type of control	Size and the oldness of the wound	No. of patients	Antibiotic application during the treatment period (if needed)	Baseline HbA1C (%)	Types of wound and grade of wound	Dressing type	Offloading	Treatment duration	Follow-up period posttherapy
[25].	RCT	Subcutaneous injection of G-CSF or saline solution for 7 days. The initial dose of G-CSF was 5 *μ*g/kg daily. The dose was lowered to 2.5 *μ*g/kg daily if, after two doses, the absolute neutrophil count was higher than 25∗10^9^/L	Placebo	>2 cm^2^ More than 8 weeks	40	Y	5·5–13·7%	NM	Standard foam dressings	NM	7 days	NM
[28]	RCT	Conventional antimicrobial treatment plus 263 mg of G-CSF subcutaneously daily for 21 days	Placebo	NM	40	Y	Y	Wagner grade III or IV	NM	Bed rest	21 days	6 months
[26]	RCT	5 *μ*g/kg G-CSF (injected subcutaneously) or placebo daily.	Placebo	0.5-3 cm	37	Y	<12%	Wagner grade II or III	NM	Bed rest	10 days	NM
[27]	RCT	Subcutaneous injection of G-CSF and/or conventional therapy. The initial daily dose of G-CSF was 5 *μ*g/kg. After three consecutive doses, if the absolute neutrophil count was >30∗10^9^/L, the dose was changed to 2.5 *μ*g/kg daily on alternate days	Placebo	NM	30	Y	NM	Wagner grade II	NM	NM	NM	NM

G-CSF: granulocyte colony-stimulating factor; Y: yes; N: no; NM: not mentioned.

**Table 8:** Outcomes of RCTs that evaluated G-CSF safety and effectiveness.

Ref	Type of growth factor	Wound closure	Mean time to heal in treatment groups	Mechanism mentioned as complete healing		Confounders				Further outcomes	
				Granulation tissue	Reepithelialization	Sex	Baseline HbA1c	Wound size	Offloading	Recurrence rate	Amputation rate
[25]	G-CSF	G-CSF therapy was associated with earlier eradication of pathogens from the infected (*p* = 0 · 02), quicker resolution of cellulitis (*p* = 0 · 03), shorter hospital stays (*p* = 0 · 02), and a shorter duration of intravenous antibiotic (*p* = 0 · 02). Neutrophil superoxide production was higher in the G-CSF-treated group (*p* < 0 · 0001)	NM	NM	NM	NM	NM	NM	NM	NM	2 cases in the placebo group
[28]	G-CSF	At the 3- and 9-week assessments, no significant differences in terms of complete closure of the ulcer without signs of underlying bone infection	NM	NM	NM	NM	NM	NM	NM	NM	15% in the G-CSF group and 45% in the control group. *p* = 0.03
[26]	G-CSF	No foot ulcer had completely healed at the end of the study. Patients who received G-CSF did not have an earlier resolution of clinically defined cellulitis (*p* = 0.57). The ulcer volume, was reduced by 59% in G-CSF and by 35% in placebo patients (*p* = 0.0005)	NM	NM	NM	NM	NM	NM	NM	NM	2 cases in total from both groups
[27]	G-CSF	No significant differences for duration of hospitalization, duration of parenteral antibiotic administration, time to resolution of infection, and need for amputation	NM	NM	NM	NM	NM	NM	NM	NM	13.3% in the treatment group and 20% in the placebo group. *p* > 0.05

G-CSF: granulocyte colony-stimulating factor; Y: yes; N: no; NM: not mentioned.

**Table 9:** Other growth factor and recombinant proteins: characteristics of RCTs.

Ref	Study	Intervention	Type of control	Size and the oldness of the wound	No. of patients	Antibiotic application during the treatment period (if needed)	Baseline HbA1C (%)	Types of wound and grade of wound	Dressing type	Offloading	Treatment duration	Follow-up period posttherapy
[29]	Phase I trial (RCT)	Topical telbermin (rhVEGF) (72 *μ*g/cm^2^) in conjunction with standard every 48 hours for up to six weeks	Placebo	1-4 cm^2^ Between 4 and 6 months old	55	Y	5.5-13.6%	Grade 1A	Covered with a sterile, semipermeable barrier and then wrapped with cotton gauze	Y	6 weeks	7-12 weeks
[30]	RCT phase IIa study	Epoetin beta injected at a weight-adjusted dose of approximately 30 IU/kg/week subcutaneously plus standard treatment	Placebo	4.09 ± 5.09 cm^2^ for a duration of 16.48 ± 18.58 months	22	NM	<8%	Wagner grade I or II	NM	Y	12 weeks	12 weeks
[31]	RCT phase I/II	2.5% or 8.5% talactoferrin gel administered topically twice daily to the ulcers with standard wound care	Placebo	0.5 to 10 cm^2^ At least 4 weeks	46	Y	6-13%	NM	Saline dressing	Y	12 weeks	Up to 6 months
[32]	RCT phase I/II	1 or 10 *μ*g Chrysalin® or saline as placebo	Placebo	0.1-8.5cm^2^ More than 8 weeks	40	Y	NM	Wagner grades I, II, or early III	NM	Y	20 weeks	NM
[33]	RCT	Five groups: standard care, topical placebo collagen sponge, or topical collagen sponge containing TGF-*β*2 either at 0.05, 0.5, or 5 *μ*g/cm^2^	Placebo	1-20 cm^2^ more than 8 weeks old	177	NM	<13%	NM	Collagen sponge and nonadherent dressing	Y	21 weeks	3 months

VEGF: vascular endothelial growth factor; TGF-*β*: transforming growth factor *β*; Y: yes; N: no; NM: not mentioned.

**Table 10:** Other growth factor and recombinant proteins: outcomes of RCTs.

Ref	Type of growth factor	Wound closure	Mean time to heal in treatment groups	Mechanism mentioned as complete healing		Confounders				Further outcomes	
				Granulation tissue	Reepithelialization	Sex	Baseline HbA1c	Wound size	Offloading	Recurrence rate	Amputation rate
[29]	Topical rhVEGF (telbermin)	A positive however nonsignificant trend towards healing in the treated group	32.5-43 days	NM	NM	NM	NM	NM	NM	27% in the VEGF group versus 33% in the placebo group	NM
[30]	Erythropoietin, epoetin beta	No significant results. 26.7% of patients receiving EPO achieved complete wound closure within 12 weeks, whereas only 14.3% in the placebo control arm	44 days	NM	NM	NM	NM	NM	NM	NM	NM
[31]	Talactoferrin gel	The active arms showed a trend toward improvement over placebo (*p* = 0.09) 33% complete healing after 30 days in the active group versus 19% in the placebo group	30 days	NM	NM	NM	NM	NM	NM	NM	NM
[32]	Chrysalin® (TP508 or rusalatide)	More than doubled the incidence of complete healing (*p* < 0.05), increased the mean closure rate 80% (*p* < 0.05), and decreased the median time to 100% closure by 40% (*p* < 0.05).	80 days to 100% closure in 10 *μ*g Chrysalin®	Y	Y	NM	NM	NM	NM	NM	1 in 1 *μ*g Chrysalin®
[33]	TGF beta2	Proportion of patients with wound closure increased in TGF-*β*2 at doses of 0.05 *μ*g/cm^2^ (*p* = 0.046) and 0.5 *μ*g/cm^2^ (*p* = 0.025) and group with standardized care treatment (*p* = 0.009). In total, the mean time to complete wound closure was shorter in TGF-*β* 0.5 *μ*g/cm^2^ (*p* = 0.03) compared with the placebo group	13 weeks in high-dose TGF beta	NM	NM	N	N	Y(-)	NM	NM	NM

VEGF: vascular endothelial growth factor; TGF-*β*: transforming growth factor *β*; Y: yes; N: no; NM: not mentioned.

**Table 11:** Adverse events related to the intervention.

Ref	Type of growth factor	Drug-related main adverse effects				
		Pain	Erythema and edema	Cellulitis	Infection	Others
[8–14, 48]	PDGF	N	N	N	N	
[21–24]	FGF	N	N	N	N	
[16–20]	EGF	N	N	N	N	Dizziness, shivering, and chills appeared more frequently in the EGF-treated groups, apparently dose-dependent
[25–28]	G-CSF	N	N	N	N	Worsened liver function, skin efflorescence
[29]	VEGF	N	N	N	N	
[30]	Erythropoietin, epoetin beta	N	N	N	N	
[31]	Talactoferrin	N	N	N	N	
[32]	Chrysalin®	N	N	N	N	
[33]	TGF-*β*	N	N	N	N	

PDGF: platelet-derived growth factor; G-CSF: granulocyte colony-stimulating factor; VEGF: vascular endothelial growth factor; TGF-*β*: transforming growth factor *β*; N: no drug-related side effect.

**Table 12:** Quality assessment based on Jadad score of randomized controlled trials reviewed.

Study	Randomization	Blinding	Account of all patients	Total score
[16]	2	2	1	5
[17]	1	1	1	3
[18]	1	0	1	2
[19]	1	1	1	3
[20]	2	2	1	5
[25]	2	2	1	5
[27]	1	0	1	2
[26]	1	1	1	3
[28]	1	1	1	3
[29]	1	1	1	3
[30]	1	1	1	3
[31]	1	1	1	3
[32]	1	1	1	3
[33]	2	2	1	5
[8]	1	1	1	3
[9]	2	1	1	4
[10]	1	1	1	3
[11]	1	0	1	2
[12]	1	0	1	2
[13]	2	0	1	3
[14]	1	1	1	3
[21]	1	0	1	2
[22]	1	0	1	2
[23]	1	1	1	3
[24]	2	2	1	5
[15]	1	0	1	2
				Mean: 3.15
				SD: 1.04
